# Close contact experiences with invasive meningococcal disease contact tracing: Implications for practice

**DOI:** 10.1177/10105395261474917

**Published:** 2026-08-07

**Authors:** Adriana Milazzo, Lynne C. Giles, Manjusha K. Sathiananthan, Scott Hanson-Easey, Louise Flood, Helen S. Marshall

**Affiliations:** 1 School of Public Health, Adelaide University, SA, Australia; 2 Robinson Research Institute, Adelaide University, SA, Australia; 3 Children’s Ground Top End, Darwin, NT, Australia; 4 Communicable Disease Control Branch, Department for Health and Wellbeing, SA Health, Government of South Australia, Adelaide, Australia; 5 Vaccinology and Immunology Research Trials Unit, Women’s and Children’s Health Network, Adelaide, SA, Australia; 6 Adelaide Medical School, Adelaide University, SA, Australia

**Keywords:** invasive meningococcal disease, infectious diseases, contact tracing, prevention

## Abstract

The aim of this study was to gain in-depth understanding of the experience of the contact tracing process by close contacts of invasive meningococcal disease (IMD) cases. Close contacts participated in semi-structured interviews. We interviewed 26 close contacts of IMD cases; most presented to a hospital emergency department for antibiotic chemoprophylaxis within 24 hours. They had prior knowledge about IMD but experienced anxiety about contracting it; many believed that the antibiotic prevented them from contracting IMD. Contacts were satisfied with information they received during the initial contact from the health department but were concerned about phone calls from an unidentified phone number being disingenuous. Most did not receive any information at the hospital about antibiotic side effects or its function. Findings from this study have implications for the public health response and management of close contacts recommended in jurisdictional IMD guidelines, for Australia and more broadly internationally.

## What We Already Know

- Contact tracing is an effective strategy in the prevention and control of invasive meningococcal disease.
- Invasive meningococcal disease contact tracing of close contacts is resource intensive.
- Definitions of close contacts of invasive meningococcal disease vary highlighting inconsistent approaches in the public health response and management.

## What This Article Adds

- Close contacts misunderstood the purpose of receiving antibiotic chemoprophylaxis.
- Busy hospital emergency departments should be avoided in sending close contacts for antibiotic chemoprophylaxis assessment.
- An alternative to unidentified telephone calls should be provided, as unknown numbers were perceived as scam calls, reducing engagement with meningococcal disease contact tracing.

## Introduction

Invasive meningococcal disease (IMD), caused by the bacteria *Neisseria meningitidis* can result in significant morbidity including meningitis or septicaemia, and for some cases, sequelae associated with infection can be severe leading to death. The IMD notification rate in Australia in 2022 was 0.5 cases per 100 000 population with genogroup B meningococci (MenB) accounting for 83% of cases.^1^ The highest proportion of notifications was in children aged <5 years (22%), and adolescents aged 15 to 19 years (23%). In South Australia (SA), the location for this study, IMD notifications have varied in recent years. In 2019, SA had the second highest notification rate at 1.5 cases per 100 000 population and the highest proportion of MenB cases in Australia.^2^ Since 2018 to 2019, SA has offered 2 meningococcal vaccination programmes for infants and adolescents: the MenACWY conjugate vaccine for eligible groups, and MenB vaccine introduced in 2018 for infants and in 2019 for adolescents.^3^

Pharyngeal carriage of *N. meningitidis* usually occurs prior to IMD. Population prevalence of asymptomatic meningococcal carriage is around 10%, with carriage rates higher in adolescents and young adults.^4^ Carrier state may be chronic, intermittent or transient, with the latter lasting several months. Risk factors for meningococcal carriage include gender (male), recent respiratory tract infection, smoking (active or passive) and low socioeconomic status.^5^

Contact tracing is an effective control measure for preventing onward transmission of some infectious diseases^6,7^ and is an essential strategy in the prevention and control of IMD. In Australia, contact tracing is the responsibility of government health departments and begins with case management to establish the timing and type of contacts prior to their onset of illness. It is particularly important to identify close contacts as they are most likely to be carriers and potentially transmit the bacteria. Management of IMD close contacts requires administration of chemoprophylaxis (clearance antibiotics) to eliminate nasopharyngeal carriage (and thus transmission) of what could be a potentially hyper-virulent serogroup circulating within their social network. Additional public health management of close contacts includes provision of information regarding IMD, so that persons act promptly if compatible clinical features occur. Vaccination is offered in some circumstances to reduce the risk of disease, and to reduce pharyngeal carriage and transmission in the unvaccinated cohort.^5^

National IMD guidelines in Australia provide definitions of close contacts (Table 1) as household/household-like, intimate kissing/sexual, child care, co-passengers and health care workers.^8^ These close contact categories and definitions, aside from the specific duration, are comparable with those used in international guidelines.^9-12^

**Table 1.** Invasive Meningococcal Disease Close Contact Definitions (7 Days Prior to the Onset of the Case’s Symptoms and Until Completion of 24 Hours of Antibiotic Treatment).

Household Household-like • Dormitory, sleep in the same household as the case
Intimate kissing/sexual
Childcare • Two full-day attendance (one full day is 6-8 hours) or cumulative of 20 hours in the same care group as the case
Co-passengers • Seated immediately adjacent to the case of >8 hours of duration
Health care workers • Exposure during airway management or resuscitation up until the case has had 24 hours of appropriate antibiotic treatment

Source: Communicable Diseases Network Australia, Australian Government Department of Health and Aged Care.

Invasive Meningococcal Disease CDNA National Guidelines for Public Health Units.^8^

Little is known about whether close contacts receive the recommended chemoprophylaxis, and adequacy of information about IMD from relevant health authorities. Studies to date have focussed on assessing policies and guidelines for the public health management of IMD,^9-12^ attitudes and barriers towards meningococcal vaccination,^13,14^ knowledge and practices regarding IMD prevention^15^ and chemoprophylaxis prescribing.^16,17^

Limited understanding of close contacts of IMD cases and their experience of the contact tracing process has implications for public health practice for prevention of IMD. In SA, all contacts of an IMD case are actively followed up by the Communicable Disease Control Branch (CDCB) at the SA Department for Health and Wellbeing (SA Health) based on national IMD guidelines.^8^ Invasive meningococcal disease cases or caregivers are interviewed to identify contacts, and to determine the type, duration and frequency of contact and until the case has received 24 hours of appropriate antibiotics. Close contacts defined by IMD national guidelines are provided with information about IMD and symptoms, recommended chemoprophylaxis and at the time of this study, SA-based close contacts were directed to the emergency department of a metropolitan tertiary hospital to receive the antibiotic/s, (or other location if the contact resided in regional SA), and advised to get vaccinated dependent on the meningococci serogroup isolated from the case. The aim of this study was to develop an in-depth understanding of the experiences of IMD close contacts during the contact tracing process.

## Methods

### Design and Setting

In-depth semi-structured interviews were conducted using questions informed by guidance from CDCB. Telephone interviews were used to gather information on participants’ relationship to the case and type of contact, adherence and satisfaction with receiving chemoprophylaxis, understanding and knowledge about IMD and reasons for chemoprophylaxis, perception about risk of disease and preference for receiving IMD information.

### Recruitment of Participants

At the time of contact tracing, close contacts were informed by CDCB staff about the study and consented to be contacted by the research team if interested in participating. The parent or guardian gave consent for child close contacts. Telephone interviews were subsequently arranged with consenting participants. Participants were recruited and interviewed between October 2019 and November 2020.

### Analysis

This study is informed by a realist theoretical framework, which assumes that social phenomena exist independently of our interpretations of them, while recognising that our knowledge of those phenomena is necessarily partial and socially mediated.^18^ Interviews were transcribed verbatim, coded, and thematically analysed using NVivo 12 software. Personal information that was potentially identifiable was pseudonymised and an identification number generated for each participant. Thematic analysis was employed to identify and interpret patterns of meaning across the dataset through data familiarisation, coding and the iterative construction and refinement of themes to develop analytic narrative.^19^ Code saturation was reached when later interviews did not generate new themes.^20^

## Results

Nine IMD cases were notified to CDCB during the study period with 105 close contacts reported, and from these 26 (24.8%) were interviewed (Table 2). Despite more close contacts agreeing to participate in the study at the time of approach, many later declined or were lost to follow-up for unknown reasons. We were not provided with demographic information on these contacts, hence cannot compare similarities and differences between participants and non-participants.

**Table 2.** Number of Close Contacts Per Case and Number Interviewed.

IMD case number	Total number of close contacts per IMD case	Number and percentage of close contacts interviewed
**Case A** *Close contacts A1 to A10*	23	10 (43.5%)
**Case B**	9	0
**Case C** *Close contacts C1 to C5*	12	5 (41.7%)
**Case D** *Close contacts D1 to D5*	25	5 (20%)
**Case E**	8	0
**Case F** *Close contacts F1 to F3*	9	3 (33.3%)
**Case G** *Close contact G1*	1	1 (100%)
**Case H** *Close contact H1*	5	1 (20%)
**Case I** *Close contact I1*	13	1 (7.7%)

Most close contacts were connected to the case through household (family) or household-like exposure (n = 16, 61.5%). Long-duration travel of a junior sporting team and affiliated adult members (n = 10, 38.5%) represented a sizeable proportion of interviewed close contacts. Most participants attended the hospital ED, except for 5 household-like dormitory close contacts (19.2%) who attended their university medical centre.

Participants were asked questions concerning demographics. Eighteen respondents provided their age (median 27, range 13-52 years). Eleven (57.9%) of 19 respondents travelled overseas and were mostly related to the junior sporting team that competed in an overseas tournament. Unvalidated vaccination status was known in 14 contacts, with 9 (64.3%) reporting that they had been vaccinated for IMD. Of the 9 IMD cases, MenB was detected in 7 cases and serogroup Y in 2 cases. Both serogroups B and Y are vaccine preventable, although we were unable to determine how many close contacts followed the advice from CDCB to receive vaccination.

Four major themes were identified with a range of sub-themes. Illustrative quotes for each of the themes are shown in Table 3.

**Table 3.** Illustrative Quotations for Identified Themes.

Theme	Illustrative quotation
Knowledge of IMD and role of antibiotics	I’ve seen a lot on the news, of course, you know. . .young babies and those horrible rashes and . . . in I guess news and media people having a terrible time and becoming incapacitated and affecting limbs and . . . all sorts of things. So . . . yeah it’s quite horrific. (A3) Just to be sure like if I was a carrier or if I already had the disease but I wasn’t showing any symptoms, just to be sure that I didn’t get it. (D2)
Compliance with public health recommendations	Look, all they really did is told us that we had to go in and have this antibiotic. They didn’t really . . . no one mentioned side effects or you know . . . they just said it was a precautionary thing to have and you know it was sort of reinforced a few times that it was you know minimal risk but that it was important to have this tablet. So, and you know, all the families are all you know they’re . . . I mean, I don’t know what, what the right thing to say is but they’re all good responsible families and you know ah, all caring, loving parents, so all the boys were in hospital over these few days getting it, yeah. (A5) Yeah, my understanding is that it was to clear any possible infection so that I can’t spread it. I think I was a bit confused about whether or not it actually protected me. That was probably more my lack of understanding rather than their lack of explaining it, so yeah. She did a good . . . I think my impression was that if I already had the disease and it had already spread but then the antibiotics could not help me. But if I hadn’t developed any symptoms then maybe the antibiotics would have helped. That’s kind of what I understood but I don’t know if that’s right, so yeah. (G1)
Perception of the quality of the contact tracing process	I guess I knew what the symptoms were and they were emailed to me as well. That was also a really good thing, like getting emailed the symptoms and watch out for it because it can develop in like 10 days, or something like that. (D3) It was mostly handy to know the symptoms and stuff as well, so I could know what I was looking out for, because a lot of my friends didn’t actually go and get the antibiotic but still lived in fairly close proximity to the person that was diagnosed. So like knowing the symptoms and knowing sort of what to keep an eye out for was good. (D4)
Experience of close contacts attending hospital for antibiotics	I tell you what, I was impressed with how efficient the system was. You know, you watch the news now and all you hear about things ramping and issues . . . with the hospital system, but on this occasion I, you know, I congratulate all of them; they did well. (A7) . . . the doctor was, he was reasonably polite but a little bit. . . abrupt with us because you know we weren’t there as an emergency I guess. And he sort of, he was a little bit rushed at the hospital because . . . he had people waiting which I understand. . .but we were just there on the advice given to us. (A3) Err yes. He said it was a once off tablet um, not to have um, which was labelled on the box as well not to have any milk product for the . . . next two hours and just the, um none of the calcium or iron supplements or anything . . . um during that period after [name of the close contact] had had the antibiotic. He said that there could be a slight risk of a bit of nausea . . . for 24-hour period which . . . (A3)

### Theme 1: Knowledge of IMD and Role of Antibiotics

#### Source of Knowledge

Participants had some prior knowledge about IMD, mostly from the media, and associated the disease with terms like “shocking” and “horrible.” Young adult close contacts recalled receiving information about IMD when they were vaccinated against it in the past, most of them at school.

At the time of contact tracing, CDCB staff provided close contacts with information (email or in person) concerning IMD and directed to a hospital for chemoprophylaxis assessment. Some close contacts disclosed that they did not read the fact sheet as they felt they received sufficient information when talking to CDCB, or they were not motivated to go through it at that time of “crisis.” Contrastingly, some participants were happy to have received information via email to refer to it later.

#### Risk of Infection

Upon knowing that they might be a close contact, most participants experienced anxiety, and worried about their risk of contracting the disease. While few close contacts felt reassured only after their hospital visit to receive chemoprophylaxis, most expressed that their concerns were allayed upon receiving information and reassurance via the initial phone call from CDCB. Contact tracing had improved their understanding about IMD, made them vigilant against signs and symptoms and more willing to seek urgent medical attention if it presents in the future.

#### Understanding the Purpose and Side Effects of Clearance Antibiotics

Many close contacts believed that clearance antibiotics was to prevent them from contracting the disease. They described it as a “preventative measure” or a “precautionary thing,” which would “protect” them from IMD. Some participants felt “pretty safe” and that they were “going to be okay” after taking the antibiotic. Similarly, one of the close contacts considered that the antibiotic reduced their risk of contracting the disease:

My understanding was that there’s a slight chance because I was in contact with a meningococcal [case] and then by taking that antibiotic it brings the chance down to, I think it was 1%, or just under 1%. (A8)

Other participants understood that the antibiotics prevented further spread of infection through them being potential carriers. Information concerning the purpose of chemoprophylaxis was not fully understood by participants, with many unaware of potential side effects. All but one close contact (who received an injection) received the antibiotic in the form of a single tablet administered orally at the hospital, with many unable to recall its name.

### Theme 2: Compliance With Public Health Recommendations

#### Time Between Initial Call From CDCB and Presentation for Clearance Antibiotics

Of the participants instructed to attend the hospital ED for chemoprophylaxis, all except one presented within 24 hours of the initial CDCB contact. The one who presented after 24 hours waited 48 to 50 hours for convenience of time and preference:

. . . we were told that it wasn’t like you had to rush in. . . . But I made the decision that . . . because our children weren’t going back to school ‘til the Tuesday, so I took [the close contact] in sort of mid-morning on the Monday, ’cause I thought it’d be quieter than the weekend. (A5)

#### Vaccination

In a follow-up call by CDCB after contact tracing, close contacts reported that they had complied with the recommendation to receive vaccination.

### Theme 3: Perception of the Quality of the Contact Tracing Process

#### Communication

Participants were satisfied with the quality of communication by CDCB. They expressed their appreciation with words like “fairly comprehensive,” “very helpful,” “comforting,” “reassuring,” “fantastic,” “did everything over and above” and “impressed with how efficient the system was.” Consensus by participants was that they were given ample opportunity to ask questions and clarify any concerns.

Most participants were satisfied with the mode of communication, but several raised the concern of receiving the call on a no-caller ID or a number unknown to the case and it being a scam call:

Probably like my only criticism is that like getting a call from a private number and like not knowing who it is, initially I like was asking a lot of questions to make sure that it wasn’t something . . . like I didn’t really sort of know what was going. (D4)

### Theme 4: Experience of Close Contacts Attending Hospital for Antibiotics

#### Wait Times

Wait times of participants attending the hospital ED for chemoprophylaxis typically ranged between 1.5 and 2 hours. Several participants had a longer wait time, with one close contact waiting for up to 3.5 hours, but none of them reported to be disappointed by the wait. The wait time for participants who visited the university medical centre was around 5 minutes or less as they had booked an appointment before visiting.

#### Service Quality

Most participants, based on self or parent-report, were happy with the service they received at the hospital, although only a few reported that they were provided with the opportunity to ask ED staff questions. When invited to ask questions, most participants requested information about the antibiotic and how long it would take to “work.” Furthermore, participants also reported seeking information regarding risk of IMD to the people they lived with or their work colleagues. Some participants were given detailed information about IMD signs and symptoms and how to respond if they begin showing symptoms, while equally others reported not receiving any information. Most participants did not receive any information on side effects or function of the antibiotic, and only one reported receiving information on the precautions to take after drug administration.

A few participants reported being rushed by the attending hospital doctor. One participant who felt rushed assumed the pandemic situation to be a possible explanation for it, “I guess with Corona going on, I could hear them talking, calling over the speaker Corona resus so I guess it might’ve been a crazy time there”. (G1) In addition, a few participants noted confusion among hospital staff regarding the protocol for chemoprophylaxis, which caused undue delay in the assessment.

## Discussion

This study provides an in-depth insight into the experiences of IMD close contacts with contact tracing. Overall, close contacts were satisfied with information provided to them by contact tracers, were compliant with receiving clearance antibiotics and attended the hospital ED within 24 hours of the initial contact from CDCB. However, their understanding of the purpose for chemoprophylaxis varied with most believing that it protected them against infection. Information from the hospital EDs was limited.

Participants in our study were mostly household or household-like contacts. Transmission of IMD largely occurs from prolonged close interaction with an asymptomatic contact with evidence showing that household contacts are potential carriers of *N meningitidis* as well as at increased risk of disease.^5^ Although the number of close contacts in our study was small, they appeared to meet the definition of a close contact according to national IMD guidelines.^8^ Correct identification of close contacts also meant they were appropriately advised to receive antibiotic chemoprophylaxis. In contrast, in other countries IMD chemoprophylaxis was advised to a wider range of contacts who did not meet national recommendations.^11^ General practitioners in the United Kingdom had overprescribed double the courses of recommended antibiotics, and in some cases legitimate close contacts did not appear to receive chemoprophylaxis.^17^ Similarly, in a Danish study, an additional 200 to 250 people were estimated to have been given chemoprophylaxis which was not recommended.^21^ An overprescribing of antibiotics for IMD contacts suggests variation from recommendations in guidelines, increasing the potential for harm and development of antibiotic resistance associated with inappropriate use.

Generally, participants felt they had good knowledge about IMD and recognised it to be a serious condition. Similarly, a study examining parent’s attitudes towards MenB vaccination found that parents recognised it to be life threatening and needing urgent medical attention.^14^ Understanding the seriousness of IMD motivated our participants to respond promptly to any advice or recommendations given by CDCB as demonstrated by 100% compliance to accessing antibiotics.

A systematic review on the effectiveness of contact tracing showed that provider-initiated tracing was associated with improved control of infectious diseases, increase in case detection, and a decrease in transmission and disease incidence. However, the findings could not identify best practice for contact tracing due to the heterogeneity of approaches taken.^22^ In our study, participants discerned that the initial telephone contact by CDCB was very helpful as it improved their understanding of IMD, highlighting the quality of contact tracing processes. Counter to this were concerns about receiving a telephone call from an unidentified number which participants thought was a scam call, potentially limiting the effectiveness of contact tracing because of privacy and confidentiality issues.^6,23^ Given participants preferred telephone contact, consideration should be given to enabling identification of telephone calls from provider-initiated contact tracers, such as sending a text message immediately prior to the call alerting contacts that the call will be from the health department.

Practices that were in place in SA before the COVID-19 pandemic, such as attending the hospital ED for chemoprophylaxis, have since changed with close contacts now directed to primary health care services. Although not evaluated in our study, attending primary health care services may be more effective with shorter wait times than in EDs. There was variability in the experience of our study participants with hospital ED processes. Participants felt that little information was provided to them by medical staff about IMD or about the antibiotics, while other participants had positive feedback about the ED experience, even in the setting of long wait times. Despite information provided to close contacts about the purpose of chemoprophylaxis by CDCB contact tracers, there was variation in their understanding. Many believed that the antibiotic was to prevent infection, which may be associated with fear of IMD and disease prevention. This finding concurs with another in which contacts, notably health care workers, were offered post-exposure vaccination although not within recommendations.^11^

### Limitations

With the implementation of public health measures in 2020 to contain COVID-19, IMD numbers in SA decreased as was experienced globally, hence our pool of IMD close contacts over the study period was small. Just over a third of the interviews were from contacts of a single case (junior sports team) and their experience to other contacts may be different. Because of this the results may not be representative of the perceptions of the contact tracing process for close contacts. Despite this, data saturation was achieved and no new codes or themes relevant to the study objectives emerged in the later interviews.

## Conclusion

Our study explored IMD close contacts’ experiences with the contact tracing process and provides evidence to inform public health decision making and guide best practice. Contact tracing is resource intensive, yet the main themes arising from the interviews highlight overall satisfaction with the quality of the process complemented by individualised follow-up and information provided to close contacts by trained tracers. While there are a wide range of contact tracing strategies including the use of digital tools,^24^ best practice recommendations highlighted in our study include providing an alternative to unidentified telephone numbers, avoiding the use of busy hospital EDs and access to simple information. Consideration should also be given to the local context, the infectious disease of concern and characteristics of contacts. Although the study was conducted in an Australian setting, our findings have relevance across jurisdictions for contact tracing at the local level. Furthermore, effective contact tracing plays a critical role to maintaining national and global health security, and enabling rapid response to infectious disease threats.
